# Stem cell lineage survival as a noisy competition for niche access

**DOI:** 10.1073/pnas.1921205117

**Published:** 2020-07-01

**Authors:** Bernat Corominas-Murtra, Colinda L. G. J. Scheele, Kasumi Kishi, Saskia I. J. Ellenbroek, Benjamin D. Simons, Jacco van Rheenen, Edouard Hannezo

**Affiliations:** ^a^Institute for Science and Technology Austria, A-3400 Klosterneuburg, Austria;; ^b^Division of Molecular Pathology, Oncode Institute, The Netherlands Cancer Institute, 1066 CX Amsterdam, Netherlands;; ^c^The Wellcome Trust/Cancer Research UK Gurdon Institute, University of Cambridge, Cambridge CB2 1QN, United Kingdom;; ^d^Department of Applied Mathematics and Theoretical Physics, Centre for Mathematical Sciences, University of Cambridge, Cambridge CB3 0WA, United Kingdom;; ^e^The Wellcome Trust/Medical Research Council Stem Cell Institute, University of Cambridge, Cambridge CB2 1QN, United Kingdom

**Keywords:** stem cell dynamics, biophysical modeling, stochastic processes, mammary morphogenesis, intestinal renewal

## Abstract

Understanding to what extent stem cell potential is a cell-intrinsic property or an emergent behavior coming from global tissue dynamics and geometry is a key outstanding question of systems and stem cell biology. Here, we propose a theory of stem cell dynamics as a stochastic competition for access to a spatially localized niche, giving rise to a stochastic conveyor-belt model. Cell divisions produce a steady cellular stream which advects cells away from the niche, while random rearrangements enable cells away from the niche to be favorably repositioned. Importantly, even when assuming that all cells in a tissue are molecularly equivalent, we predict a common (“universal”) functional dependence of the long-term clonal survival probability on distance from the niche, as well as the emergence of a well-defined number of functional stem cells, dependent only on the rate of random movements vs. mitosis-driven advection. We test the predictions of this theory on datasets of pubertal mammary gland tips and embryonic kidney tips, as well as homeostatic intestinal crypts. Importantly, we find good agreement for the predicted functional dependency of the competition as a function of position, and thus functional stem cell number in each organ. This argues for a key role of positional fluctuations in dictating stem cell number and dynamics, and we discuss the applicability of this theory to other settings.

## Significance

What defines the number and dynamics of the stem cells that generate and renew biological tissues? Although several molecular markers have been described to predict stem cell potential, we propose a complementary approach that mathematically describes “stemness” as an emergent property arising from a stochastic competition for space. We predict from that competition the robust emergence of a region made of functional stem cells, as well as give simple predictions on lineage-survival probability. We test our results with data obtained from intravital live-imaging experiments in mammary gland development, existing data from kidney development, and from the self-renewal of the crypt to show that our framework can predict the number of functional stem cells and lineage-survival probability.

Many biological tissues are renewed via small numbers of stem cells, which divide to produce a steady stream of differentiated cells and balance homeostatic cell loss. Although novel experimental approaches in the past decade have produced key insights into the number, identity, and (often stochastic) dynamics of stem cells in multiple organs, an outstanding question remains as to whether stem cell potential is a cell-intrinsic, “inherited” property or, rather, an extrinsic, context-dependent state emerging from the collective dynamics of a tissue and cues from local “niches,” or microenvironments (1–8). Although recent experiments have provided evidence for the latter in settings such as the growing mammary gland (9), adult interfollicular epidermis (10, 11), spermatogenesis (12), or the intestinal epithelium (13), a more global theoretical framework allowing one to quantitatively interpret these findings is still lacking.

The case of the intestinal crypt serves as a paradigmatic example of the dynamics of tissue renewal and is one of the fastest in mammals (13). The intestinal crypt consists of a small invagination in the intestine where the epithelial cells populating the intestinal walls are constantly produced. The very bottom of the crypt hosts a small number of proliferative, Lgr5+ stem cells (14) that divide and push the cells located above them to the transit amplification (TA) region, where cells lose self-renewal potential. Cells are eventually shed in the villus a few days later, constituting a permanent “conveyor-belt” dynamics. Lineage-tracing approaches, which irreversibly label a cell and its progeny (3), have been used to ask which cell type will give rise to lineages that renew the whole tissue and have revealed that all Lgr5+ cells can stochastically compete in an equipotent manner on the long-term (15–18), but still display positional-dependent short-term biases for survival (13). Interestingly, similar conclusions have been reached in pubertal mammary gland development (9), where branching morphogenesis occurs through the proliferation of the cells in the terminal end buds of the ducts (19), the region where the mammary stem cells (MaSCs) reside (9, 20). In both cases, intravital imaging revealed random cellular motions, enabling cells to move against the cellular flow/drift defined by the conveyor-belt dynamics. Moreover, in the intestine, tissue damage, or genetic ablation of all Lgr5+ stem cells, caused Lgr5– cells to recolonize the crypts and re-express Lgr5 to function as stem cells (13), arguing for extensive reversibility and flexibility in the system (21). In addition, Lgr5– and Lgr5+ cells of the fetal gut were also shown to nearly equally contribute to intestinal morphogenesis (22). Altogether, this supports proposals that the definition of stem cell potential should evolve to emphasize, instead of molecular markers, the functional ability of cells to renew over the long-term (23, 24).

However, this definition raises a number of outstanding conceptual problems: What, then, defines the number of functional stem cells in a tissue? How can short-term biases be reconciled with long-term equipotency? Is there a sharp distinction between stem and nonstem cells, or is there instead a continuum of stem cell potential together with flexible transitions between states? Qualitatively, it is clear that fluctuations and positional exchanges are needed to prevent a single cell in the most favorable position to be the unique “functional” stem cell (defined as cells whose lineage colonizes a tissue compartment on the long-term). Incorporating these features in a dynamical model of stem cell growth and replacement, able to make predictions, e.g., on the probability of lineage perpetuation, would represent an important step toward the understanding of how stem cells operate in the process of tissue growth and renewal.

**Fig. 1.**
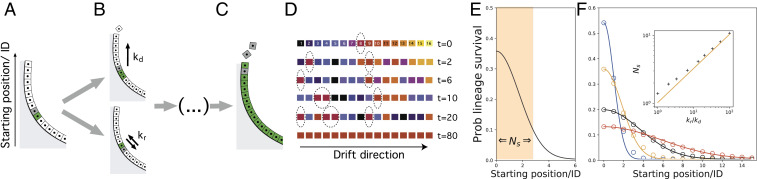
SCB as a paradigm for stem cell renewal. (*A* and *B*) A cell in the epithelial wall of the crypt (*A*) can duplicate at rate $k_{d}$, pushing the upper cells up, creating a conveyor-belt mechanism, or switch its position randomly at rate $k_{r}$, introducing a stochastic or noisy ingredient in the dynamics (*B*). (*C*) At longer time scales, the lineage of a single starting cell colonizes the whole system. (*D*) Example of SCB dynamics. At t=0, we have N=16 lineages in the system, depicted with different colors and at starting positions 1,…,16, respectively. In time, lineages are progressively eliminated, but stochastic cell rearrangements make it possible for a lineage far from the origin (starting position n=8 in red and highlighted with a dashed circle) to win the competition. (*E*) Probability (prob) that a given lineage colonizes the entire system as a function of initial position of its mother cell, decaying as a Gaussian of width $\sqrt{k_{r}/k_{d}}$; see *Dynamics of Tissue Renewal and Development* for details. The width of this distribution defines a functional stem cell region ($N_{s}$ cells, highlighted in orange, plotted for $k_{r}/k_{d}=3$). (*F*) Numerical simulations of the one-dimensional SCB dynamics. We compute the long-term survival probability $p\left(c_{n}\right)$ as a function of initial starting position n=0,1,2,…, with respect to the base of the system for several values of $k_{r}/k_{d}$ (1, 3.3, 13.3, and 33 in, resp., blue, orange, black, and red). Dots show the outcome of the simulations, and lines show the analytical prediction $p\left(c_{n}\right)∼\exp\left\{-\frac{k_{d}}{2k_{r}}n^{2}\right\}$, as shown in Eq. **4**. *F*, *Inset* shows the plot of best fit for the variance of the numerical distributions (black crosses) against the analytical model prediction $∼\sqrt{k_{r}/k_{d}}$ (orange solid line).

In this paper, we develop a reaction–diffusion formalism for stem cell renewal in the presence of noise and local niches, taking into account local tissue geometry, as well as cell division and random cell movements (Fig. 1 *A*–*C*). Importantly, within this purely extrinsic and dynamical approach, which does not need to posit any intrinsic “stem cell identity,” a well-defined number of functional stem cells emerges, which only depends on the geometry and a balance between the noisiness of cell movements and division rates advecting cells away from niche regions. This model also predicts that stem cell potential should decay continuously as a function of distance from the niche, with a “universal” Gaussian functional dependence. We test this prediction against published live-imaging datasets for the homeostatic intestinal crypt (13) and during the branching of embryonic kidney explants (25) and find a good quantitative agreement for the full survival probability of cells, depending on their initial position relative to the niche. Furthermore, we use our theoretical results to extract the amplitude of the random positional fluctuations in the developing mammary gland using static lineage-tracking experiments (9). This enables us to predict the number of functional stem cells for this system, finding values consistent with previously reported estimates.

## Dynamics of Tissue Renewal and Development

To develop the model, we first considered the simplest situation of a one-dimensional column of cells, with a rigid boundary condition at the base (mimicking, for instance, the bottom of the crypt), so that each cell division produces a pushing force upward transmitted to the cells above (or, in the case of growing mammary gland or kidney, driving ductal elongation). This model is motivated by its simplicity, as it is able to qualitatively derive the essential traits of the complex dynamics studied here. As we shall see, further refinements, aimed at making predictions for real systems, considered more realistic geometries. From this simple dynamics, we defined the number of functional stem cells as the typical number of cells that have a nonnegligible probability to produce long-term progenies (without “losing” the competition against other cells). If the dynamics was fully devoid of noise (a simple conveyor belt) and all cell divisions were symmetric, then one of the bottom-most cells would always win the competition. In the case of a one-dimensional array of cells, this problem is trivial. If one considers a cylindric geometry, there would be a single row of functional stem cells, which is the limiting case of the model described in ref. 16 of symmetric and stochastic one-dimensional, neutral competition along a ring of equipotent cells. However, live-imaging studies show that, in multiple settings, including mammary gland (9), kidney morphogenesis (25, 26), and intestinal crypts (13), there is widespread rearrangement of cells through stochastic cell movements (27). Intuitively, such rearrangements are expected to increase the number of “functional” stem cells, as rearrangements allow cells away from the niche to relocate to favorable positions, and would thus provide a biophysical mechanism for setting the number of stem cells assumed in models such as that developed in ref. 16.

The simplest abstraction of the system is a one-dimensional column of N cells. Each cell divides at constant rate $k_{d}$. In one dimension, we assumed a rigid boundary at the bottom so that cell proliferation generated a net flow of cells along the positive axis, i.e., advection away from the niche. In addition, the position of the cells can fluctuate stochastically at rate $k_{r}$ (either via local cell–cell rearrangements, or more global movements of cells relative to the niche; *SI Appendix*, sections S1A and S4), allowing cells far away from the niche to reposition, despite the overall flow.

At t=0, each cell is characterized by its starting position n (distance from the niche) and will give rise in time to a lineage denoted $c_{n}$, which can span the entire tissue. However, as soon as a cell reaches the position N, it disappears from the system, resulting after a sufficiently large time period in a single surviving lineage. This competitive dynamics can be metaphorically understood as a conveyor belt with random fluctuations in the cell positions, sketched in Fig. 1 *A*–*C*. This is why we call it *stochastic conveyor belt* (SCB) dynamics and use it to model tissue renewal (e.g., intestinal crypt homeostasis) or organ growth (e.g., kidney and mammary gland morphogenesis). The only difference between these two general cases is a change of reference frame (*SI Appendix*, section S1 and Fig. S1). In Fig. 1*D*, we show an example of a typical run of the simulated SCB dynamics in one dimension, until monoclonality is achieved (see also *SI Appendix*, Movies S1–S3 and section S5A for details).

To make quantitative predictions from the dynamics outlined above, we start by following the prevalence of a single lineage. Here, the action of the other lineages can be imposed as an average drift force that depends on the position of each cell of the lineage we follow. The equation accounting for the time evolution of the prevalence of lineage $c_{n}$, to be referred to as $\rho_{n}\left(z,t\right)$, in the continuum limit is:

$$\frac{\partial \rho_{n}}{\partial t}=-k_{d}\frac{\partial }{\partial z}\left(z\rho_{n}\right)+\frac{k_{r}}{2}\frac{\partial^{2}\rho_{n}}{\partial z^{2}}+k_{d}\rho_{n}.$$ [1]

We refer to this reaction–diffusion equation (28, 29) as the SCB equations (see *SI Appendix*, section S1B for details). The first term on the right-hand side is a drift term, accounting for the average push-up movement at position z due to random cellular proliferation at rate $k_{d}$ at lower levels, $∼k_{d}z$. The second term is a diffusive term (30, 31), accounting for the random reallocations of cells, occurring at rate $k_{r}$. The third term is a proliferative term, accounting for the exponential proliferation of each cell of the lineage under study, at rate $k_{d}$.

Considering initial conditions $t_{0}=0$, $\rho_{n}\left(z,0\right)$, a Gaussian centered on n with $\sigma^{2}=1/2$ (a density representing a single cell at position n), and natural boundary conditions, the solution of Eq. **1** can be approximated by (see *SI Appendix*, section S1B for details):

$$\rho_{n}\left(z,t\right)\approx \sqrt{\frac{k_{d}}{2\pi k_{r}}}\exp\left\{-\frac{k_{d}}{2k_{r}}{\left(\frac{z-ne^{k_{d}t}}{e^{k_{d}t}}\right)}^{2}\right\}.$$ [2]

Next, we sought to relate this lineage prevalence to the experimentally relevant quantity of long-term lineage survival; in other words, how likely is it for a cell starting at a given position n to take over the entire crypt? Although lineage fixation is a concept that only makes sense in the discrete description, we observed that lineage prevalence converges asymptotically toward a simple scaling form $\rho_{n}\left(\infty \right)$:

$$\rho_{n}\left(\infty \right)\equiv \underset{t\to \infty }{\lim}\rho_{n}\left(z,t\right),$$ [3]

which is a constant that does not depend on position z or time t, but only on the starting position of the lineage. This argues that, on the long-term, lineages starting at different positions n and n′ have well-defined relative prevalence, leading to the natural assumption that the long-term lineage-survival probability of lineage $c_{n}$ is proportional to this asymptotic lineage prevalence. This means that the probability of lineage survival, $p\left(c_{n}\right)$, can be expressed as:

$$p\left(c_{n}\right)\approx \frac{\rho_{n}\left(\infty \right)}{\sum_{j}\rho_{j}\left(\infty \right)}\propto \exp\left\{-\frac{k_{d}}{2k_{r}}n^{2}\right\}.$$ [4]

The above equation, which is a central result of the study, defines the probability that a cell starting at position n will “win the competition” and colonize the whole one-dimensional system (see *SI Appendix*, section S1 for details).

Despite the approximations outlined above, stochastic numerical simulations of the model system show excellent agreement with Eq. **4** (Fig. 1 *E* and *F*). We also note that, although we have assumed here that positional rearrangements occur between two cells, more complex sources of positional noise $k_{r}$ can be considered (which can be mechanistically dependent or independent on $k_{d}$) and lead to the same qualitative results. These include, for instance, postmitotic dispersal, as seen during the branching morphogenesis of the kidney uteric bud (26) and where daughter cells can travel long distances outside the epithelium postdivision, or correlated “tectonic” movements of the epithelium, where cells could collectively reposition relative to the niche, as proposed during mammary or gut morphogenesis (9, 22) (see *SI Appendix*, section S4 for details).

## Functional Stem Cell Numbers and Dynamics in the SCB

The prediction for the probability of long-term lineage survival under the SCB dynamics is surprisingly simple, decaying as a Gaussian distribution as a function of position away the niche, with a length scale that is simply the amplitude of the stochastic fluctuations divided by the proliferation rate, $∼\sqrt{k_{r}/k_{d}}$ (Eq. **4**). Intuitively, cells close to the origin have the highest chance to win and survive, whereas this probability drops abruptly for cells starting the competition further away, i.e., around $N_{s}$ cell diameters away from the base, with:

$$N_{s}^{1D}=1+\sqrt{\frac{k_{r}}{k_{d}}}.$$ [5]

Note that the first term satisfies the boundary condition that, in the case $k_{r}=0$, the system has a single functional stem cell (located at the base) in one dimension. Eq. **5** thus implies that multiple rows of cells possess long-term self-renewal potential (as assessed, for example, in a lineage-tracing assay), emerging through their collective dynamics, and with a number that depends only on the ratio of the division to rearrangement rates (respectively [resp.], $k_{d}$ and $k_{r}$). Although Eq. **5** is the outcome of a one-dimensional approximation, we show that it holds and can be generalized in more complex geometries (*SI Appendix*, section S3). In particular, in a cylindrical two-dimensional geometry, we show that the functional stem cell number would simply be the same number $N_{s}^{1D}$ of cell rows (arising from the SCB dynamics) multiplied by the number of cells per row (fixed by the geometry of the tissue). Moreover, the above result can be generalized, giving an estimate of $N_{s}$ for general geometries (see *SI Appendix*, Eq. **26**, where we give the general expression for $N_{s}$ in arbitrary organ geometries). This general result will be at the basis of the forthcoming sections, when dealing to more realistic geometries to explore the dynamics of the organs under study. Importantly, our framework generalizes the work of ref. 16, as we do not fix the stem cell number $N_{s}$ explicitly, which, rather, emerges from an interplay between geometry and SCB dynamics, together with the competitive dynamics being qualitatively different in the flow direction (*SI Appendix*, section S1).

We now turn to experimental data to test whether the proposed dynamics can help predict the number of functional stem cells in several organs, as well as the evolution of the survival probability with the starting position of a clone. Although the division rate $k_{d}$ is well known in most systems considered, the stochastic movement rate $k_{r}$ is harder to estimate and can potentially vary widely, from rather small in intestinal crypts (13) to large in mammary and kidney tips, with extensive clonal fragmentation and random cell movements (9, 25).

### Predictions on Clonal Dynamics and Survival.

**Fig. 2.**
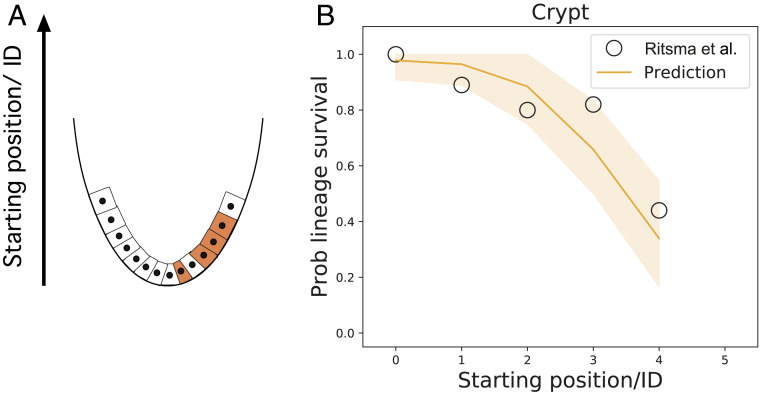
(*A*) Schema of the self-renewal of the crypt epithelia, showing the origin of the coordinate system at the bottom of the system. (*B*) The probability (prob) that a given lineage remains within the system as a function of the starting position after a time lapse against the predictions of the conveyor belt dynamics for the crypt. Data corresponding to the probability that a lineage remains in the system for the small intestinal crypt, reported in Ritsma et al. (13), depending on its starting position. The orange line represents the prediction of the SCB dynamics, fitting well the data for $k_{r}/k_{d}\approx 1$. Shaded areas represent the CI (one SD) of the prediction.

Intravital live imaging provides an ideal platform to test the model, as it provides both knowledge of the starting position of a given cell, as well as its clonal time evolution (whereas classical lineage tracing relies on clonal ensembles obtained from fixed samples). In small intestinal crypts, different Lgr5+ cells have been predicted to have very different lineage-survival potential on the short-term, depending on their position within the stem cell niche, resulting in an effective number of stem cells smaller than the number of Lgr5+ cells (13, 32). We thus reanalyzed quantitatively this dataset by plotting the survival probability of a clone as a function of its starting position n (Fig. 2) after a given time period assumed to be large enough for Eq. **4** to hold. We then compared this to a two-dimensional stochastic simulation of the model (see *SI Appendix*, section 5 for details). Importantly, we found a good qualitative and quantitative agreement between model and data, with the survival probability decaying smoothly with the starting position (Fig. 2*B*). The only parameter here was $k_{r}/k_{d}\approx 1$, which fits well with short-term live-imaging experiments and the idea of cell division promoting rearrangements (13).

To back these simulations with an analytical prediction on stem cell numbers, the details of tissue geometry must be taken into account (with the number of cells per row i needing to be estimated and the number of rows participating in the competition arising as an emergent property from the one-dimensional model). A good approximation is based on that fact that the crypt can be abstracted as a hemispherical monolayer with radius R (measured in units of cell diameter) coupled to a cylindrical region (see *SI Appendix*, Figs. S1, S3, and S4 and section S3 for details), so that one can get the number of stem cells, $N_{s}^{2D}$, as:

$$N_{s}^{2D}\approx 2\pi R^{2}\left[1-\cos\left\{\frac{1}{R}\left(1+\sqrt{\frac{\pi }{2}\frac{k_{r}}{k_{d}}}\right)\right\}\right].$$ [6]

With $k_{r}/k_{d}\approx 1$ as above, and estimating R≈2 for the radius, our simple theory then predicts that the number of functional stem cells should be $N^{2D}\approx 11$, which agrees well with measurements of ref. 13, as well as inferred numbers from continuous clonal labeling experiments (32). This is expected, as our model reduces to the one-dimensional ring model of ref. 16 for low $k_{r}/k_{d}$.

We then sought to test the model further using a published dataset on embryonic kidney branching in explants (25). This has been recently noted to be a highly stochastic process, with neighboring cells at the start of the tracing ending up either surviving long-term in tips or being expelled to ducts. Moreover, ref. 25 observed extensive random cell intercalations, in addition to the described mitotic dispersal (26), where cells extrude from the epithelium postdivision and reinsert at a distance of $d_{c}$ cell diameters away. Importantly, these processes can still be captured as an effective diffusion coefficient $k_{r}$ in our framework (see *SI Appendix*, section S4 for details). Specifically, knowing that the fluctuations may occur at each duplication, and that they imply a displacement up to $d_{c}\approx 2-4$ cell lengths, we can estimate that $k_{r}/k_{d}\approx d_{c}^{2}\;$ at the minimum (i.e., discounting other fluctuations). Note that the conveyor-belt dynamics applies exactly for tip elongation as in crypt: The only difference is that the reference frame from which the dynamics is observed changes (see *SI Appendix*, section S1 and Fig. S1 for details).

**Fig. 3.**
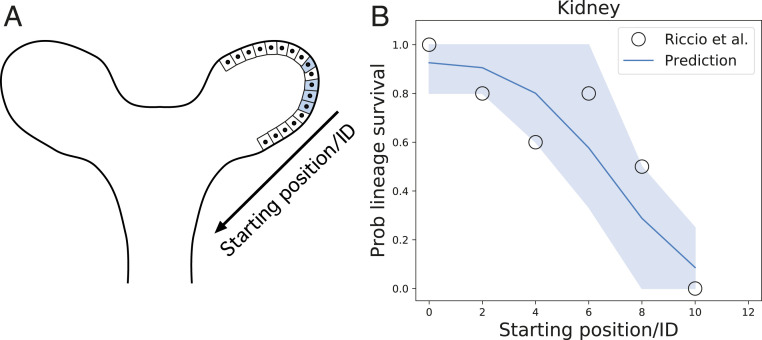
(*A*) Schema of the kidney tip during development. The conveyor-belt dynamics holds; the only difference is the reference frame: Whereas in the stem cell replacement model of the intestinal crypt, the reference frame is the bottom of the gland, in the kidney and mammary gland, the reference frame is taken from the newly created ducts. (*B*) The probability (prob) that a given clonal remains within the system as a function of the starting position of the mother cell after a given time against the predictions of the conveyor-belt model dynamics. Black circles represent real data points, obtained by counting the amount of cells of a given lineage remaining in the system [from Riccio et al. (25)]. We observe that the distribution is much broader, fitting well to the theory for a ratio $k_{r}/k_{d}\approx 16$ in kidney, over an order of magnitude larger than in intestinal crypt. The shaded area represents the CI (one SD) of the prediction.

The above observation argues again that noise will play a key role in kidney-tip cell dynamics. Strikingly, extracting from ref. 25 the probability of survival as a function of distance from the edge of a tip, we found that the two-dimensional simulations of our model provided again an excellent prediction for the full probability distribution (Fig. 3), with cells much further away (compared to the intestinal crypt) having a nonnegligible probability to go back and contribute. Again, the only fit parameter was the ratio $k_{r}/k_{d}=16$, which agrees well with our estimate of the noise arising from mitotic dispersal. Taking into account the full two-dimensional geometry as above, and estimating in this case a tip radius of R=3−5 cells, this predicts $N_{s}\approx 90\pm 10$, which could be tested in clonal lineage-tracing experiments.

These two examples show that the same model of SCB dynamics and its prediction of the master curve for the survival probability of clones can be used in different organs to understand their stem cell dynamics and that ratios of relocation to advection $k_{r}/k_{d}$ can be widely different, even in systems with similar division rates $k_{d}$.

### Number of Functional Stem Cells in the Developing Mammary Gland.

**Fig. 4.**
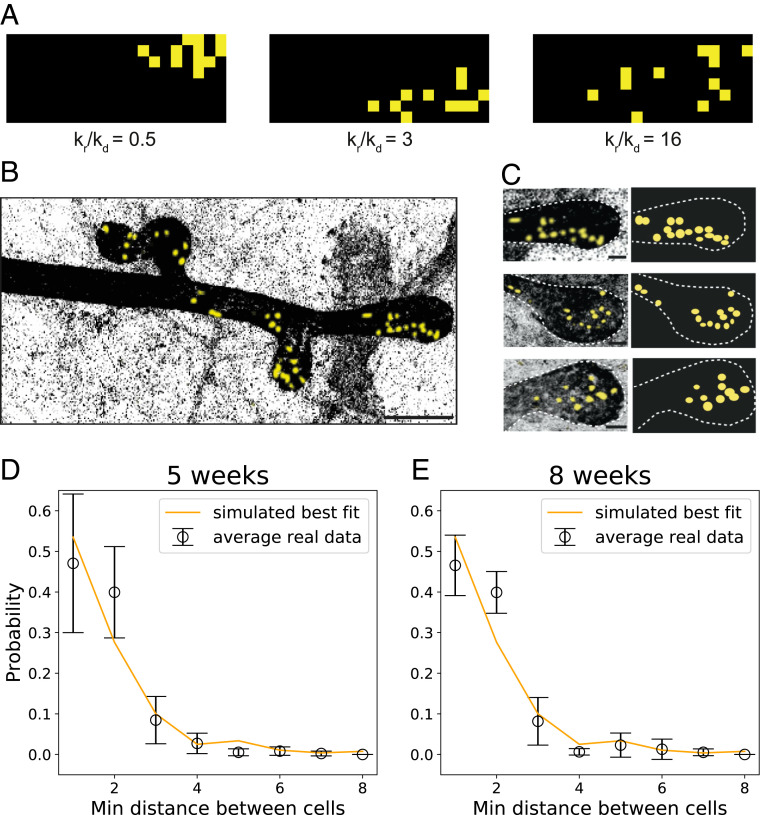
(*A*) Inferring the relation $k_{r}/k_{d}$ from the clone dispersion using a simulation of the SCB dynamics in two dimensions. The distribution of distances of the closest neighbors is highly sensitive to the relation $k_{r}/k_{d}$. Here, we show numerical simulations of fragmentation under increasing (left to right) values of $k_{r}/k_{d}$. (*B*) Growing tips of a developing mammary gland together with sparse lineage-tracing experiments, where a single lineage (yellow here, induced in 3-wk-old animals) can be observed. Clonal dispersion due to random cell rearrangements is observed. (*C*) Close-up of three different mammary tips (*Left*) and corresponding reconstructions to extract relative cellular positions (*Right*). The geometry of the end buds can be approximated by a hemispherical structure connected to a cylindrical one, whose radius can be inferred to be around two to five cell diameters. (*D* and *E*) Probability distributions of nearest distances between clonally related cells in tips (resp., from 5- and 8-wk old mice). Black dots represent experimental data (basal and luminal cells have been treated together for this analysis, as they do not show different behavior at the level of the dynamics). Orange lines are from two-dimensional numerical simulations of the SCB model (see *SI Appendix*, *SI Text* for details), showing a good fit from $k_{r}/k_{d}\approx 3$ for both time points. Error bars represent mean and SD. Scale bars represent 100 μm (*B*) and 10 μm (*C*). Min, minimum.

Next, we sought to test the suitability of the SCB dynamics to model stem cell dynamics of mammary gland morphogenesis, where extensive cell movements have been reported within tips via intravital live imaging (9), with rapid rearrangements occurring on time scales of a few hours (Fig. 4 and *SI Appendix*, Fig. S6*A*). In this case, however, tips cannot be followed for long enough for survival probabilities to be directly measured as in Figs. 2 and 3 for intestine and kidney, respectively. However, extensive clonal dispersion has been observed in quantitative clonal lineage-tracing experiments during pubertal growth (9, 33), and we therefore sought to infer the value of noise from these experiments (*SI Appendix*, Fig. S6*B*).

Turning back to published lineage-tracing datasets, where single MaSCs are labeled at the beginning of puberty (3 wk of age) and traced until either 5 or 8 wk of age, clones in tips displayed extensive fragmentation, which is expected to be directly related to the ratio $k_{r}/k_{d}$ (Fig. 4 *C* and *D* and *SI Appendix*, Fig. S6 *B*–*D*). We thus ran as above two-dimensional simulations of our SCB dynamics (see *SI Appendix*, section S5 for details), using measured values of the tip width and length to set the geometry. As a metric for clonal dispersion, we then computationally measured for each labeled cell the distance to its closest clonal neighbor: For a fully cohesive clone, all cells should be touching, and the distance to the closest neighbor should be always one cell diameter. Increasing the value of $k_{r}/k_{d}$ robustly increased the closest neighbor distance. We then performed the same measurements in the experimental dataset, both for the 5- and 8-wk time points (Fig. 4 *C* and *D*) and also for luminal and basal cell types separately, given the dominant unipotency of these cell populations in pubertal development (9, 33–35). We found highly consistent results in all four cases (average closest distance of around 1.85 cell diameter), which allowed us to infer a ratio of (see *SI Appendix*, section S5 and Fig. S6 for details):

$$k_{r}/k_{d}\approx 2-5,$$ [7]

in mammary gland, emphasizing the importance of considering stochasticity in the conveyor-belt picture. Indeed, we found that, with this fitting parameter, the model reproduced well the probability distribution of closest distances, both at the 5- and 8-wk time points (Fig. 4 *C* and *D*).

In addition to this value, we must again pay attention to the geometry of the mammary tip, with basal cells forming a two-dimensional monolayer (similar to the previous cases), while luminal cells form multiple layers in three dimensions within the tip. Assuming that the intercalation between cells occurs mainly at the same layer, the system of luminal cells in the tip of the mammary gland can be abstracted as R−1 successive hemispherical two-dimensional layers. Let us emphasize the dependence of $N^{2D}$, as defined by Eq. **6**, on R, writing $N_{s}^{2D}\equiv N_{s}^{2D}\left(R\right)$. In that case, the amount of luminal stem cells can be inferred as:

$$N_{s}^{3D}=\underset{R\prime <R}{\sum }N_{s}^{2D}\left(R\prime \right).$$ [8]

Taking the fitted range of $k_{r}/k_{d}\in \left(2,5\right)$, together with an estimation of the radius of R=5±2, Eq. **8** then predicts that a number of luminal stem cells per tip of $N_{s}^{3D}=170\pm 110$, in good quantitative agreement with experimental estimates from lineage tracing of $N_{s}^{\exp}=172\pm 102$ (mean ± SD) (9). For basal cells, using the same parameters for a two-dimensional monolayer, Eq. **6** predicts that $N_{s}^{2D}=37\pm 11$, against empirical observations reporting an amount of basal stem cells of at least 15 (33), and $N_{s}^{\exp}=93\pm 76$ (mean ± SD) (9). Although the prediction thus falls in the correct range, the underestimation of basal stem cell number may be due to the highly anisotropic geometry of basal stem cells.

## Discussion

The main objective of this study was to provide insights to the question of whether stem cell function is a cell-intrinsic, inherited property, or rather an extrinsic, context-dependent notion emerging from the collective dynamics of a tissue (2, 3, 7, 8). To that end, we took a complementary standpoint to the one based on the classification of molecular markers and their potential functional role, adopting a purely dynamical/geometrical descriptions of niches. Combining the two would be a logical extension for future work. We analyzed stem cell lineage survival as a purely dynamical process of competition for finite niche space, taking into account the presence of stochastic cell rearrangements, cell proliferation, and tissue geometry. This gives rise to a complex reaction–diffusion process that can be abstracted as an SCB. We show that survival probability as a function of starting position away from the most favorable position adopts a simple universal Gaussian shape, so that a well-defined number of functional stem cells (i.e., cells which have a nonnegligible probability of surviving long-term) arises in the theory, set by tissue geometry and the ratio between random reallocation and cell proliferation rates, $k_{r}/k_{d}$. We applied this theory to recent live-imaging data tracing stem cell survival as a function of position in the homeostatic intestinal crypt and kidney morphogenesis and found good quantitative agreement. We also used the model to infer values of $k_{r}/k_{d}$ from fixed lineage-tracing experiments in mammary-gland morphogenesis and showed that this inference allows us to predict the typical number of stem cells in this system. Interestingly, the ratio of noise to advection $k_{r}/k_{d}$ appeared to be an order of magnitude larger in kidney development as compared to the intestinal crypt (with the mammary gland being intermediate), which explained well the widely different number of functional stem cells observed in each.

Although we have sketched here the simplest source of noise in cellular movements (random exchange of position in cell neighbors), our results are highly robust to different types of microscopic mechanisms and should thus be seen as representative of a general class of models for stem cell dynamics with advection and noise. In mammary-gland and kidney morphogenesis, direct cell–cell rearrangements are observed (9, 25), while kidney also displays mitotic dispersal (26), where noise arises from the randomness of cell reinsertion in the layer after division. Furthermore, on short time scales, directed cellular movements have been observed in kidney-tip morphogenesis, with Ret and Etv4 mutant clones being statistically overtaken by wild-type cells, leading to the proposal that Ret/Etv4 were involved in directional movement toward tips (25). However, tips maintain heterogeneity in Ret expression through branching, arguing that cells must shuttle between high-Ret and low-Ret states (25), which would effectively contribute to movement stochasticity on long time scales. Finally, “tectonic” movements, which collectively reposition cells toward/away from niches, can also be captured in the model (*SI Appendix*, Fig. S5). These are particularly relevant in developmental settings, such as gut morphogenesis, where the global shape of the epithelium changes, displacing collectively cells from villus to crypt regions (22), or upon tip-splitting during branching morphogenesis (9). Active migration, as observed in adult intestinal homeostasis (36), could also contribute to such collective random repositioning events. In the future, it would be interesting to further understand quantitatively random cell rearrangements $k_{r}$ and how they could be modulated by parameters such as tissue density, aspect ratio, active cell migration, or division rates (see *SI Appendix*, section S3C for details). Mechanical models of cell motility upon rheological transitions (37–39) or of rearrangements and junctional remodeling upon cell divisions (40, 41) in densely packed tissues could also help to understand quantitatively what sets $k_{r}$ in each system.

The proposed framework can, in principle, be applied to any tissue dynamics in which niche signals and/or cellular proliferation is localized, leading to directional flows (42, 43). On the other hand, substantial extension of the model would be necessary in the context of an “open niche,” such as spermatogenesis (44) or skin homeostasis (11), where renewing cells form a two-dimensional layer of neutrally competing progenitors, thus with little in-plane cellular flows. Finally, the theory could be extended to cases of nonneutral growth. Live imaging of skin-tumor growth, for instance, is consistent with very low values of $k_{r}/k_{d}$ (45), as little to no clonal dispersion is observed, which would tend to favor deterministic growth in our model. Nevertheless, this does not occur, as tumor cells trigger higher proliferation rates of normal cells (45), resulting in complex geometrical changes and encapsulation of the malignant clone. Incorporating these types of complex signaling and geometric feedbacks between multiple cell populations (46–48) in our model would thus have particular relevance to understand the dynamics of tumor initiation (49, 50). Our approach must be taken as part of a more general enterprise, namely, understanding the role of both intrinsic cues and complex collective dynamics in defining the functional stem cells.

## Materials and Methods

Additional information on the theoretical, computational, and experimental methods used can be found in *SI Appendix*, *SI Materials and Methods*. In brief, all mice for mammary-gland experiments were females from a mixed background, housed under standard laboratory conditions. All experiments were performed in accordance with the Animal Welfare Committee (Instantie voor Dierenwelzijn) of the Royal Netherlands Academy of Arts and Sciences (Hubrecht Institute). Clonal dispersion in the developing mammary tips was measured in whole-mount glands from R26-CreERT2;R26-Confetti mice, traced from 3 wk of age and killed at midpuberty (5 wk) or at the end of puberty (8 wk). The length and the width of the tips were measured, and the coordinates of each labeled confetti cell in the tip were determined, to calculate the distance between each cell and their closest neighbor. Raw data used to generate Fig. 4 can be found in *SI Appendix*, Dataset S1.

## Supplementary Material

Supplementary File

Supplementary File

Supplementary File

Supplementary File

Supplementary File
