# Supplementary material for: Stem cell lineage survival as a noisy competition for niche access
Source: Proc Natl Acad Sci U S A. 2020 Jul 1;117(29):16969–75. doi: 10.1073/pnas.1921205117 (PMC7382312; doi:10.1073/pnas.1921205117)
Supplement: Supplementary File [file pnas.1921205117.sapp.pdf]

PNAS
www.pnas.org

Supplementary Information for

Stem cell lineage survival as a noisy competition for niche access

B. Corominas-Murtra, C. L.G.J. Scheele, K. Kishi, S. I.J. Ellenbroek, B. D. Simons, J. van Rheenen and E. Hannezo

[2]To whom correspondence should be addressed. E-mail: bernat.corominas-murtra@ist.ac.at, j.v.rheenen@nki.nl, edouard.hannezo@ist.ac.at

This PDF file includes:

Supplementary text
Figs. S1 to S6
Legends for Movies S1 to S3
SI References

Other supplementary materials for this manuscript include the following:

Movies S1 to S3

www.pnas.org/cgi/doi/10.1073/pnas.1921205117

Supporting Information Text

1. Basic dynamics

The simplest abstraction of our system is composed by a 1-dimensional column of cells arranged in a finite segment $[0, N]$, in which the length unit is scaled to the average length of a cell. In addition, in this 1-dimensional setting, it is necessary to additionally assume that at 0 there is a rigid wall. Each cell divides at constant rate k_d. Due to the rigid boundary at the bottom, cell proliferation generates a net flow of cells towards the positive axis. In addition, the position of the cells can fluctuate stochastically at rate k_r (either via local cell-cell rearrangements, or more global movements of cells relative to the niche, see sections below for more details). At $t = 0$, each cell defines a lineage, labeled by the position they occupy, i.e., the cell located at position n at $t = 0$ will be the *mother* of the lineage c_n. From here on, due to proliferation, drift and random reallocations, we can potentially find cells of lineage c_n in all regions of the organ. However, as soon as a cell reaches the position N and is pushed, it disappears from the system, eventually resulting in the disappearance of a whole lineage from the system. Notice that, if more than one lineage is present, for a sufficiently long time, the probability of lineage loss is always greater than zero. Since the lineage loss is an irreversible event, that means that one expects that the system will sooner or later become monoclonal, meaning that a single lineage populates the whole system. This competitive dynamics can be metaphorically understood as a conveyor belt with random fluctuations in the cell positions, sketched in Fig. 1 of the main text. This why we call it *Stochastic Conveyor Belt* (SCB) dynamics.

Both intestinal crypts and elongating mammary or kidney tips can be described by the SCB dynamics, if we consider that it develops over more realistic geometries, such as hemispheric surfaces. An hemispheric surface has the coordinate origin at the bottom/top pole (the tip in the kidney and mammary gland buds, and the bottom of the crypt), and there is no need to assume rigid boundaries anywhere, as the symmetry of the geometry will trigger the SCB dynamics, producing a net flow of cells towards the positive axis z, which defines the direction the organ grows/renews. Qualitatively, the dynamics can be abstracted in the same way in the three organs, up to a change of referential frame: intestinal crypts expel differentiated cells with the crypt base staying stationary, while differentiated cells stay in place while the tip moves forward in the case of mammary gland or kidney development –see figure (S1). Importantly, the results that we derive, as described below, are generic to different types of assumptions on the microscopic dynamics, as long as the basis features of advection and rearrangements exist in a system (see sections below for details and examples).

A. Lineage prevalence evolution. A full model of the lineage dynamics would require keeping track of each lineage as cell divide and re-arrange (given the constraint that only one cell can occupy a given position of the tissue), leading to an extremely intertwined dynamical system where a few or no analytical predictions can be derived. To overcome this problem, we first make a mean-field approximation, whose suitability will be validated in subsequent section from numerical simulations. We consider a continuous array of cells of length N in which each point (cell) is subject to the SCB dynamics. Given a position x, the effect of proliferation at lower levels is captured by an average (mean-field) push-up term, $\sim k_d x$, enabling us to decouple the evolution of different lineages and derive analytical predictions for the prevalence of a given lineage (i.e. cells arising from an ancestor which started at position n) in the tissue. We characterize the presence of cells of a given lineage c_n at a given point of the interval $[0, N]$ by a prevalence function $\rho_n(x, t)$ (analogous to an average density). To construct the equation accounting for the time evolution of the prevalence of lineage c_n, we need to consider:

- *Drift term* accounting for the mean field push up movement at position x due to proliferation at rate k_d at lower levels:

  $$-k_d \frac{\partial}{\partial x} \left(x \rho_n(x, t) \right) \quad ,$$

  since, at positions $[0, ..., x)$, $k_d x$ new cells will be produced in a time step pushing the cells at position x an average of $k_d x$ positions upwards.

- *Diffusive term* accounting for the random reallocations of cells occurring at rate k_r:

  $$+\frac{k_r}{2} \frac{\partial^2}{\partial x^2} \rho_n(x, t) \quad .$$

- *Proliferative term* accounting for the exponential proliferation of the cells of the lineage under study at rate k_d:

  $$+k_d \rho_n(x, t) \quad .$$

Combining these terms, the equation accounting for the time evolution (in units of $\tau = k_d t$) of such prevalence is:

$$\frac{\partial \rho_n}{\partial \tau} = -\frac{\partial}{\partial x}(x\rho_n) + \frac{k_r}{2k_d} \frac{\partial^2 \rho_n}{\partial x^2} + \rho_n \quad . \qquad \text{[S1]}$$

We will refer to this equation as the *SCB equation*.

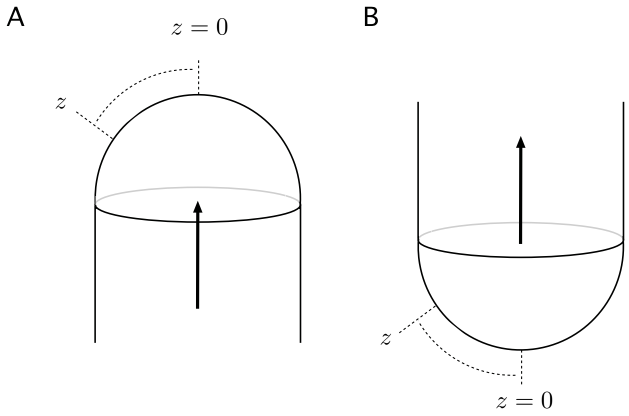

Fig. S1. The SCB dynamics can describe several process of development and renewal, up to a change on referential frame. A/ In the case of both the mammary gland and kidney development, the organ generates new cells via tip growth while ductal cells remain stationnary, leading to relative tip movement. B/ In the case of the self-renewal of the crypt, the bottom of the system is stationary and cells are flowing upwards

B. Solutions of the SCB equation. To solve equation (S1) we impose as initial condition that $\rho_n(x,0) \sim \mathbf{N}(n,1/2)$, i.e., a normal distribution centred n with variance $1/2$. This condition describes a single cell located at position n as a density that spreads significantly only at $n \pm 1/2$ and whose integral is equal to 1, as it is expected for a single cell. Natural boundary conditions apply*. We observe that the solution of equation (S1) factorizes as follows:

$$\rho_n(x,t) \propto \phi_1(x,t)\phi_2(t) \quad , \tag{S2}$$

where ϕ_1 is the solution of:

$$\frac{\partial \phi_1}{\partial \tau} = -\frac{\partial}{\partial x}(x\phi_1) + \frac{k_r}{2k_d}\frac{\partial^2 \phi_1}{\partial x^2} \quad , \tag{S3}$$

and ϕ_2 is the solution of:

$$\frac{\partial \phi_2}{\partial \tau} = \phi_2 \quad .$$

which has a simple solution:

$$\phi_2 \propto e^{\tau} \quad . \tag{S4}$$

In words, ϕ_2 represents the fact that cells proliferate randomly at rate k_d so that the entire lineage increases in size exponentially regardless of its position.

Computing ϕ_1 is a little bit more entangled. To start with, first observe that (S3) is a Fokker-Planck like equation for the time evolution of the probabilities of a random variable following a mixture of Brownian motion with a given amplitude k_r/k_d and a drift parameter $x\phi_1(x,\tau)$. If, instead of looking at the evolution of the probabilities we look at the behaviour of the random variable itself –the position in the system, X–, equation (S3) has its stochastic differential equation counterpart in:

$$dX = Xd\tau + \sqrt{\frac{k_r}{k_d}}dW \quad , \tag{S5}$$

being dW the differential of the standard Brownian motion (1, 2). Equation (S5) describes the movement of a single cell in the SCB dynamics. Eq (S5) explicitly tracks the real trajectory of a cell (and not lineage prevalence). The above described stochastic process has no stationary solutions, which is in agreement with the SCB dynamics: all cells will sooner or later be pushed out from the system (in the case of the crypt, or will be left behind, as in the case of the mammary gland development). Imposing the following initial conditions $t_0 = 0$, $\phi_1(x,0) \sim \mathbf{N}(n,1/2)$ and natural boundary conditions, we observe that:

$$d\left(e^{-\tau}X(\tau)\right) = dX(\tau)e^{-\tau} - e^{-\tau}X(\tau)d\tau \quad .$$

Then, multiplying both sides of equation (S5) by $e^{-\tau}$, and after some algebra, one finds that:

$$d\left(e^{-\tau}X(\tau)\right) = \sqrt{\frac{k_r}{k_d}}e^{-\tau}dW \quad ,$$

leading to:

$$X(\tau) = x_0e^{\tau} + \int_0^{\tau}\sqrt{\frac{k_r}{k_d}}e^{(\tau-s)}dW \quad . \tag{S6}$$

The integral is a standard stochastic integral with respect to a Wiener process. According to *Ito's isometry* (3) one has that the law governing the random variable described by the integral is a normal distribution $\mathbf{N}(0,\sigma^2(\tau))$. In our case this reads:

$$\int_0^{\tau} e^{(\tau-s)}\sqrt{\frac{k_r}{k_d}}dW \sim \mathbf{N}\left(0, \int_0^t \left|\sqrt{\frac{k_r}{k_d}}e^{(t-s)}\right|^2 ds\right) \quad ,$$

which means that the explicit form of $\sigma^2(\tau)$, is thus given by:

$$\sigma^2(\tau) = \int_0^{\tau}\left|\sqrt{\frac{k_r}{k_d}}e^{(\tau-s)}\right|^2 ds = \frac{k_r}{2k_d}\left(e^{2\tau}-1\right) \quad . \tag{S7}$$

Finally, from equation (S6), we conclude that the time dependent mean, $\mu(\tau)$, is:

$$\mu(t,x_0) = x_0e^{\tau} \quad .$$

That leads to:

$$\phi_1(x,\tau) \propto \sqrt{\frac{k_d}{2\pi k_r\left(e^{2\tau}-1\right)}}\exp\left\{-\frac{k_d}{2k_r}\frac{(x-ne^{\tau})^2}{(e^{2\tau}-1)}\right\} \quad . \tag{S8}$$

*A rigorous approach to this problem would require a reflecting boundary condition at $x = 0$. Imposing such boundary condition would make the whole problem much more difficult and, eventually intractable. The reason by which we adopted natural boundary conditions is due to the fact that the dynamics in this system is extremely imbalanced and runs essentially in only one direction. If one takes equation (S8) at $x = 0$ we observe that the probability of being at $x = 0$ decays as $\sim e^{-\tau}$, for any starting point $n > 0$, as it is the case in our system. This tells us that the probability of visiting regions $x < 0$ is, to our purposes, negligible.

In other words, the solution is given by a random variable following a normal distribution whose mean and variance run exponentially fast in time through the positive axis. In Fig. (S2a) of this SI, we plotted some snapshots of this time dependent probability.

According to equations (S2, S4) and (S8), the solution of the SCB equation (S1) can be fairly approximated as:

$$\rho_n(x,\tau) \propto \sqrt{\frac{k_d}{2\pi k_r}} \exp\left\{-\frac{k_d}{2k_r}\left(\frac{x-ne^{\tau}}{e^{\tau}}\right)^2\right\} \quad . \qquad \text{[S9]}$$

C. Lineage survival probability. Experimentally, a measurable quantity of key interest is the long-term fixation probability ("how likely is it for a cell starting at a given position n to take over the entire crypt?"). Indeed, at a discrete cellular level, a given lineage can disappear from the conveyor belt (absorbing boundary condition at the end of the belt), so that a single lineage will be present in the crypt on the long term, something that by definition cannot be captured in the continuum model.

However, we note that the lineage prevalence from the continuum model converges on long time scales towards a simple scaling law

$$\begin{aligned} \rho_n(\infty) &\equiv \lim_{\tau\to\infty} \rho_n(x,\tau) \\ &= \sqrt{\frac{k_d}{2\pi k_r}} e^{-\frac{k_d}{2k_r}n^2} \quad , \end{aligned} \qquad \text{[S10]}$$

which is independent of x and t (see Fig. (S2b) of this SI), and dependent only of the ratio kr/kd and the starting position k. This argues that on the long-term, lineages starting at different positions n and n' have well-defined relative prevalence. Together with the observation that conveyor belts tend to monoclonality, it is then reasonable to make the assumption that

- The long term lineage fixation/survival probability is proportional to the asymptotic lineage prevalence $\rho_n(\infty)$

so that the probability of lineage survival, $p(c_n)$, can be derived directly from the normalization of the asymptotic prevalences $\rho_n(\infty)$ as:

$$p(c_n) \approx \frac{\rho_n(\infty)}{\sum_j \rho_j(\infty)} \quad ,$$

which leads to:

$$p(c_n) \propto \exp\left\{-\frac{k_d}{2k_r}n^2\right\} \quad . \qquad \text{[S11]}$$

In words, we predict that the lineage fixation/survival probability is described by a Gaussian-like distribution defined only over the positive axis with mean 0 and variance $\sqrt{\frac{k_r}{k_d}}$. Importantly, numerical simulations of the full discrete SCB model in 1 dimension revealed excellent agreement with Eq. (S11), validating our assumptions.

2. SCB dynamics in more general geometries

In general we will assume that there is a coordinate z over which the displacement induced by proliferation takes place (in practice this would be dictated by the boundary conditions, e.g. the geometry of the region where cell loss occurs). All the dynamics will be, in consequence, studied from its projection over this coordinate –see Fig. (S3) of this SI for the special cases of hemispheric and spheric geometries. In the case of a 1-dimensional system, as the one described above, this coordinate is the length, x. In the case of a hemisphere, assuming that the push-up force is exerted from the bottom pole, this coordinate is the arc length defined from the position of the cell to the bottom pole itself –see Fig. (S3a) of this SI. To gain intuition, consider the surface of the hemisphere with radius R: The cells at the bottom pole divide and push the ones on top of them up through the surface. The cell under consideration is located at a position defining an arc from the bottom pole equal to $z = R\varphi_n = z_n$, where φ_n is the polar angle, meaning that there is an arc of n cells from the given cell to the pole of the hemisphere –see Fig. (S3a) of this SI. The successive divisions of cells located at $z_i < z_n$ will result into a net displacement along the polar coordinate φ of the cell located initially at $z_n = R\varphi_n$, going from $z_n = R\varphi_n$ to $z_{k'} = R\varphi'_n$, with $\varphi'_n > \varphi_n$. The linear displacement along the surface will be $\Delta z = R(\varphi'_n - \varphi_n)$. Displacements along the other coordinate will have no net effect in the push up force.

A. SCB equation in general geometries.

A.1. Drift term. The drift term will be described by the function $h(z)$, accounting for the local speed of a cell at position z:

$$h(z) \equiv \frac{dz}{dt} \quad . \qquad \text{[S12]}$$

In the case of a 1-dimensional system, as the one described by equation (S1), one has that $h(x) = k_d x$.

To properly study this dynamics over more general geometries, let us consider a Riemannian manifold equipped with a metric tensor $\mathbf{g}$, with components g_{ij} (6). Crucial to our purposes is the property of *local flatness* (6, 7). Roughly speaking, this implies that, for small enough regions of the manifold, the geometry has euclidean properties. Let us consider that the push up force due to proliferation has an origin and is exerted along the direction of a single coordinate z as well. As we did above,

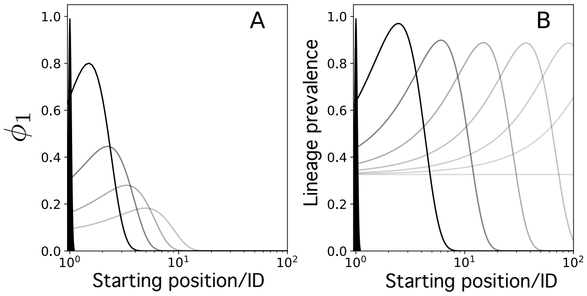

Fig. S2. Temporal evolution of the SCB dynamics (black to grey indicates time). A/ Evolution in time of the probability for cells starting at a given position n (in that case, $n = 1$) to occupy the location z at a given time according to the theoretical prediction given by equation (S8). Observe that the dynamics does not run to a stationary state, so all cells will eventually abandon the system with probability 1 as long as time grows. These probability densities refer to real probabilities that the cell is in a given position during the stochastic trajectory defined to the SCB dynamics, in contrast to the relative interpretation of the lineage prevalence. B/ Evolution in time of the prevalence of a lineage starting in the same position across all positions, according to the solution of the SCB equation (S1) given in equation (S9). Observe that the reaction diffusion dynamics displays a front that runs exponentially towards the outside of the system. Notice the prevalence reaches a non-zero stationary value, that is assumed to be proportional to the probability of the lineage to remain and colonize the whole system. The initial black, elongated triangle at position 1 shows the initial conditions i.e., $n = 1$, and $k_r = 1$, $k_d = 1$. These values have been chosen only for the sake of clarity.

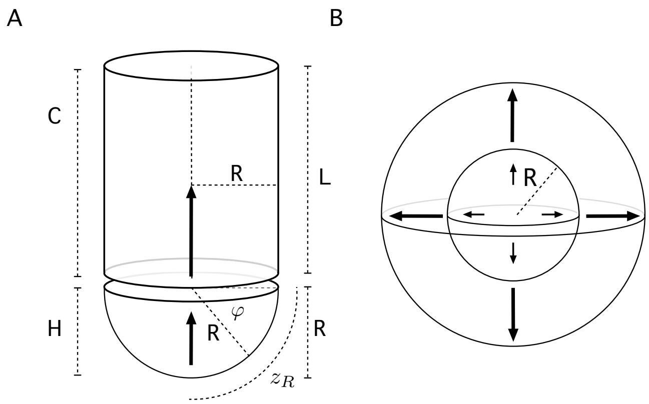

Fig. S3. A/ Schematic characterization of the structure of the crypt as a hemispherical region H coupled to a cylinder region of the same radius, R. B/ The expansion of the tissue in a 3-dimensional abstract setting where there is radial symmetry. The growing of the inner cells creates a push up force. In addition, the stochastic fluctuations in the position determine the probability of lineage survival as a function of the starting point, as in the case of low dimensional approaches.

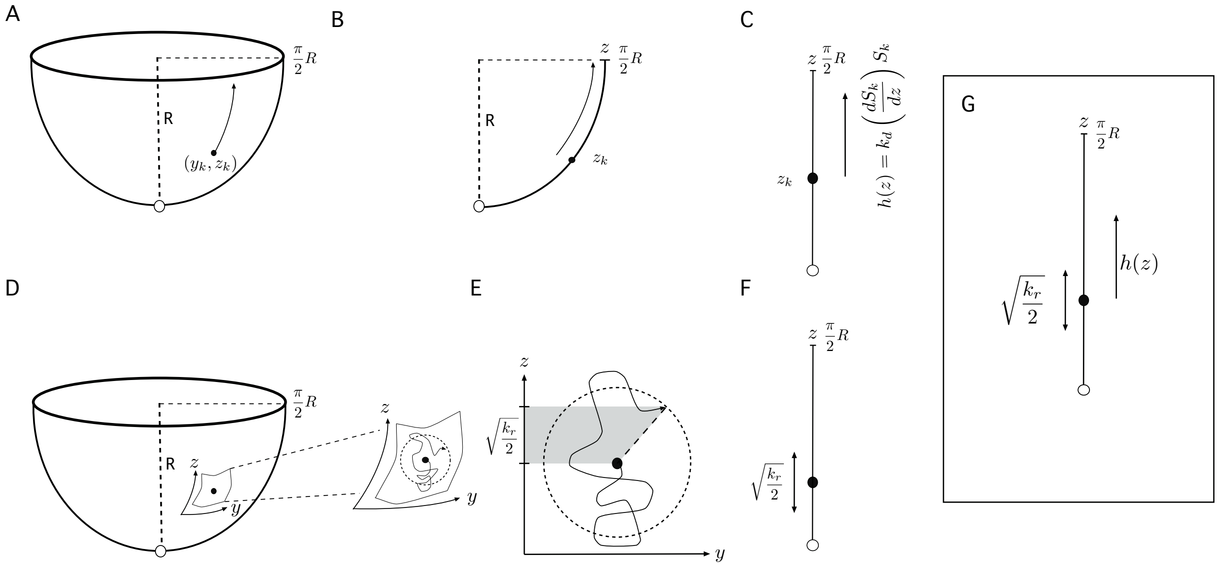

Fig. S4. Constructing the dynamical equation for a general kind of manifold and projecting it onto the 1-dimensional coordinate system along which the push-up force is exerted. A/ A cell is located in a point in a manifold –in that case, a hemisphere–, described by two orthogonal coordinates (y_n, z_n). In this case $y_n = R\theta_n$, where θ is the azimuthal angle, and $z_n = R\varphi_n$, where φ is the polar angle. B/ the push up force due to proliferation is exerted only along the z coordinate. C/ $h(z)$ is the drift term that enters the equation, and refers to the amount of new surface that has been created below the point z_n in the z coordinate that results in pushing up the cells above. D/ the same point (y_n, z_n) also observes fluctuations due to random noise. In particular, the rate of these fluctuations is externally reported as k_r. E/ Thanks to the local flatness property, if $\sqrt{k_r} \ll R$, then the fluctuations take place locally in a flat space, and the average distance from the starting point, after a unit time interval, will be $\sqrt{k_r}$. Since the coordinate system y, z is orthogonal and locally flat, and the random fluctuations occur isotropically in space, the projection of the fluctuations over the coordinate y, k_r^y, will be the same than in the coordinate z, k_r^z. Since $\sqrt{k_r} = \sqrt{k_r^y + k_r^z}$, the only solution for this projection is that, $k_r^z = k_r/2$, as described in F/. G/ Combining C/ and F/ we have that the growing process can be described as a SC dynamics along the z coordinate with drift term $h(z)$, diffusion term $\frac{k_r}{2}$, and proliferative term $\rho_n z$.

the surface/volume units are given such that an average cell has a surface/volume of 1 in the corresponding units. Consider the starting position of our cell to be z_n along the coordinate z. If the other coordinates of the manifold are $x_1, ..., x_{n-1}$, the surface/volume encapsulated *below* position z_n is given by:

$$S_n = \int ... \int_0^{z_n} \sqrt{g} dx_1 ... dx_{n-1} dz \quad ,$$

where g is the determiner of the metric tensor, i.e.:

$$g = \begin{vmatrix} g_{11} & g_{12} & . & . & . \\ g_{21} & g_{22} & & & \\ . & & . & & \\ . & & & . & \\ . & & & & . \end{vmatrix}$$

Cells are assumed to divide at rate k_d. That implies that $k_d S_n$ new cells will be produced *below* the cell located at z_n. This will create an extra surface/volume of:

$$\frac{dS_n}{dt} = k_d S_n \quad ,$$

that will project into the coordinate z. Using that:

$$\frac{dS_n}{dt} = \frac{dS_n}{dz}\frac{dz}{dt} \quad ,$$

and, then, equation (S12), one can find the general expression for this projection, which reads:

$$h(z) = k_d \left(\frac{dS_n}{dz}\right)^{-1} S_n \quad . \qquad [S13]$$

A.2. Diffusion term. We are only interested on the projection of the dynamics over the coordinate z along which the system grows, as in the other coordinates the competition is neutral and has no net effect in the lineage survival statistics. Under the assumption of local flatness, if the global stochastic reallocation rate is isotropic one can estimate the projection of such reallocations over the coordinate z, k_r^z as:

$$k_r^z \sim \frac{k_r}{D} \quad , \qquad [S14]$$

where D is the dimension of the manifold.

Let us consider in detail the 2-dimensional case: Imagine that we report experimental, isotropic fluctuations of amplitude k_r (see Fig. (S4)) of this SI. That is, in a time unit, the cells move randomly over the manifold k_r steps. We have a 2-dimensional isotropic dynamics taking place over a locally flat (7) surface with generic orthogonal coordinates y, z (for example, $R\times$ the azimuthal angle θ and $R\times$ the polar angle φ over a sphere surface). The amplitude of the fluctuations after time t is known to be $\sim \sqrt{k_r t}$, a distance defined over the surface. In the case we consider the projection over the coordinate z, thanks to the local flatness, assuming that $\sqrt{k_r} \ll R$ and using only symmetry reasonings, one has that since the displacement is given by $(\Delta y, \Delta z) = (\sqrt{k_r^y}, \sqrt{k_r^z})$:

$$\sqrt{(\sqrt{k_r^y})^2 + (\sqrt{k_r^z})^2} = \sqrt{k_r} \quad .$$

Since the fluctuations are isotropic:

$$k_r^z = k_r^y \quad ,$$

leading to:

$$k_r^z = \frac{k_r}{2} \quad . \qquad [S15]$$

In the case we are dealing with a spherical surface, we are projecting the fluctuations over the polar angle $z = R\varphi$ (see Fig. (S4) of this SI).

A.3. SCB equation. According to the above results, we will have that the general equation for the evolution of cell lineage prevalences along the coordinate z will read:

$$\frac{\partial \rho_n}{\partial t} = -\frac{\partial}{\partial z}(h(z)\rho_n) + \frac{k_r^z}{2}\frac{\partial^2 \rho_n}{\partial z^2} + \rho_n \quad . \qquad [S16]$$

In the case $h(z)$ can be approached as a linear function, i.e., $h(z) \sim a k_d z$ and k_r^z as $k_r^z = b k_r$, one can apply the assumption presented in section 1C and rewrite equation (S11) as:

$$p(c_n) \propto \exp\left\{-\frac{a}{2b}\frac{k_d}{k_r} n^2\right\} \quad . \qquad [S17]$$

B. SCB dynamics in realistic geometries. Let us now consider a detailed version of the geometry of the organs under study in the main text (mammary and kidney tips, or intestinal crypts). They are described as a half sphere, H, whose arc length from the bottom pole to the end is is $z_R = \frac{\pi}{2}R$ coupled to a cylinder C of length L and radius R. The push-up force is directed towards the top of the cylinder (see Fig. (S3a)) of this SI. In the arc that goes from the bottom pole to the end of the hemisphere there are z_R cells. Again, the units are given considering the average size of the cell as the length/surface/volume unit. Therefore, the cells will be labelled in terms of the geodesic distance over the hemisphere to the bottom pole.

B.1. Hemispheric region. The prevalence of the lineages will be given by the $\approx \frac{\pi}{2}R$ cells that populate the arc length that goes from the bottom pole of the hemisphere to the equator, where the system is coupled to the cyclinder. Let us label them as:

$$(\rho_0, \rho_1, \rho_2, ..., \rho_{z_{R-1}}, \rho_{z_R}) \quad .$$

Since the coordinate R is constant, the only dynamically relevant information will come from the angle φ. Each position n in the arc $(0, 1, 2, ..., z_n, ..., z_R - 1, z_R)$ describing the initial point of a cell lineage can be rewritten as:

$$z_n = R\varphi_n \ , \quad \varphi_n \in \left(0, \frac{\pi}{2}\right) \quad .$$

i.e., $\varphi_n = \frac{z_n}{R}$. The metric tensor for this hemispheric surface is (7):

$$\mathbf{g} = \begin{pmatrix} R^2 & 0 \\ 0 & R^2\sin^2(\varphi) \end{pmatrix} \quad .$$

Computing the determiner of $\mathbf{g}$, g:

$$g = \begin{vmatrix} R^2 & 0 \\ 0 & R^2\sin^2(\varphi) \end{vmatrix} = R^4\sin^2\varphi \quad ,$$

one can compute the surface element as (7):

$$dS = \sqrt{g}d\theta d\varphi \quad .$$

In consequence, the area under the position of the cell n in the in the hemisphere H, located at the arc position z_n, will be:

$$S_n^H = \int_0^{2\pi}\int_0^{\frac{z_n}{R}} \sqrt{g}d\theta d\varphi = 2\pi R^2\left(1 - \cos\left(\frac{z_n}{R}\right)\right) \quad .$$

By direct application of equation (S13), we have that the push-up force inside the hemisphere H is given by:

$$h^H(z) = k_d R\left[\frac{1-\cos\left(\frac{z_n}{R}\right)}{\sin\left(\frac{z_n}{R}\right)}\right] \quad . \qquad \text{[S18]}$$

Finally, from equation (S15) we know that (see also Fig. (S4) of this SI):

$$k_r^z = \frac{k_r}{2} \quad ,$$

leading, according to the SCB equation (S16) for $0 \le z \le \frac{\pi}{2}R$ in a hemispherical surface to be:

$$\frac{\partial \rho_n}{\partial t} = -k_d R\frac{\partial}{\partial z}\left(\frac{1-\cos\left(\frac{z_n}{R}\right)}{\sin\left(\frac{z_n}{R}\right)}\rho_n\right) + \frac{k_r}{4}\frac{\partial^2\rho_n}{\partial z^2} + \rho_n \quad . \qquad \text{[S19]}$$

The above equation is difficult to deal with. However, we observe that in the region of interest, $z \in \left[0, \frac{\pi}{2}R\right]$, equation (S18) can be approximated as:

$$\tilde{h}(z) \sim \frac{2k_d}{\pi}z \quad , \qquad \text{[S20]}$$

leading to an error bounded as:

$$\max_{z\in\left[0,\frac{\pi}{2}R\right]}\left|\left|h(z) - \frac{2k_d}{\pi}z\right|\right| < 0.09k_d R \quad ,$$

according to numerical tests. With this approximation, we have that SCB equation for hemispheric surfaces (S19) can be rewritten approximately as:

$$\frac{\partial \rho_n}{\partial t} \approx -\frac{2k_d}{\pi}\frac{\partial}{\partial z}(z\rho_n) + \frac{k_r}{4}\frac{\partial^2\rho_n}{\partial z^2} + \rho_n \quad , \qquad \text{[S21]}$$

which is the general kind of SCB equations we have been working so far.

B.2. Coupling to a cylinder. In the case the cell is at the position n in the cylindric region C, the area under it will be given by $S^H_{\frac{\pi}{2}R}$, the area of the whole hemisphere, and the remaining surface due to the cell is the cylinder. Knowing that for the cylindric coordinates $\sqrt{g} = R$, then:

$$S^C_n = S^H_{\frac{\pi}{2}R} + \int_0^{2\pi} d\theta \int_{\frac{\pi}{2}R}^{z_n} \sqrt{g}dz = S^H_{\frac{\pi}{2}R} + 2\pi R\left(z_n - \frac{\pi}{2}R\right) \quad .$$

Completing the picture, the push force felt by a cell in the cylindric region C is given by:

$$h^C(z) = k_d\left(z_n + \left(1 - \frac{\pi}{2}\right)R\right) \quad . \tag{S22}$$

It is easy to check that:

$$\begin{aligned}\lim_{z\to\frac{\pi}{2}R^+} h^H(z) &= \lim_{z\to\frac{\pi}{2}R^-} h^C(z)\\ \lim_{z\to\frac{\pi}{2}R^+} \frac{d}{dz}h^H(z) &= \lim_{z\to\frac{\pi}{2}R^-} \frac{d}{dz}h^C(z) \quad .\end{aligned}$$

Therefore, one can define a function, $h(z)$ as:

$$h^{H,C}(z) = \begin{cases} h^H(z) \text{ if } z \leq \frac{\pi}{2}R \\ h^C(z) \text{ if } z > \frac{\pi}{2}R \end{cases} \quad , \tag{S23}$$

which is always well defined. In addition, the projection of the fluctuations will be the same in both regions, since the only relevant property is the local dimension, which in both cases is 2, leading to a $k^z_r = k_r/2$. Consequently, equation (S16) can be rewritten consistently for all the hemisphere/cylinder system as:

$$\frac{\partial \rho_n}{\partial t} = -\frac{\partial}{\partial z}(h^{H,C}(z)\rho_n) + \frac{k_r}{4}\frac{\partial^2 \rho_n}{\partial z^2} + \rho_n \quad . \tag{S24}$$

B.3. Lineage survival probability in hemispheric geometries. We then estimate the probability of lineage survival in the limit where most of the cells with non-vanishing long-term survival probability are located in the hemispheric region H of the crypts (putting an upper bound of k_r/k_d). As discussed in section 2 of this SI, the existence of linear functions approximating the drift and fluctuation parameters of the general reaction-diffusion equation (S16) leads to Gaussian-like lineage survival probabilities. According to equation (S21) and the assumption on lineage survival probabilities presented in section 1C, we have that, considering only the hemispheric region of the crypt:

$$p(c_n) \propto \exp\left\{-\frac{2}{\pi}\frac{k_d}{k_r}n^2\right\} \quad . \tag{S25}$$

B.4. Higher dimensionality with radial symmetry. We next consider the case in which the tissue has radial symmetry (i.e. can be abstracted as a manifold isomorphic to a disk or sphere). In consequence, we will only consider the r coordinate away from the center of the tissue (see Fig. (S3b) of this SI). In the case of isotropic stochastic reallocations occurring at rate k_r, following the same reasoning we used in section 2, we can approximate the effective rate projected towards the r axis by:

$$k^r_r \sim \frac{k_r}{D} \quad ,$$

where D is the dimension of the manifold. The diffusion term in the reaction diffusion equation will be given by:

$$\sim \frac{k_r}{D}\frac{\partial^2 \rho_n}{\partial r^2} \quad ,$$

Now we compute the drift term. For that, we apply directly equation (S13). In the case of a 3-dimensional sphere growing from the centre, we have, if $V(r)$ is the volume encapsulated by the surface of radius r, the displacement of a cell at this position will be determined by:

$$\frac{dV(r)}{dt} = k_d V(r) \quad ,$$

If $V(r) = \frac{4}{3}\pi r^3$, then, according to the above equation:

$$\frac{dV(r)}{dt} \propto r^2\frac{dr}{dt} \quad .$$

Thus, the push up force will, accordingly, result in a radial displacement of:

$$\frac{dr}{dt} \propto k_d r \ , \quad h(r) \propto k_d r \quad .$$

The above speed will define the drift term $h(r)$.

According to the above results, the SCB equation for a spherical system ($D = 3$) dividing through the coordinate r will be:

$$\frac{\partial \rho_n}{\partial t} = -\frac{\partial}{\partial r}(k_d r \rho_n) + \frac{k_r}{6}\frac{\partial^2 \rho_n}{\partial r^2} + \rho_n \quad .$$

where there must be a boundary region where the cells do not proliferate anymore or a $R_{\max}$ beyond which cells are lost, in order to balance this proliferative flux.

3. Number of stem cells emerging from the SCB dynamics

The existence of Gaussian-like distributions in lineage survival probabilities enables us to compute the size of the emerging stem-cell region as the number of cells that are at a distance $\leq \sigma$ from the origin. The reason is that they represent the set of cells having the highest chances to colonize the whole system. In consequence, the origin of stem cell potential in this model is purely dynamical, and the functional stem cell number will arise simply from the interplay between geometry and dynamics.

A. General case. Consider that, as we did above, that tissues can be abstracted as a n-dimensional $(x_1, ..., x_{n-1}, z)$ Riemannian manifold equipped with a metric tensor $\mathbf{g}$ where the local flatness property holds. Assume there is an origin from which the push up force due to proliferation is exerted along the positive direction of a single coordinate z, Eq. (S17) applies, and a functional stem cell is defined as any cells within a distance σ of the niche center, meaning that, there is a *belt* containing

$$\sim \sigma + 1$$

cells from the origin of coordinate z to the last cell that is inside the stem-cell region, with:

$$\sigma \sim \sqrt{\frac{b}{a}\frac{k_r}{k_d}} \quad ,$$

Therefore, the number of stem cells emerging from the SCB dynamics in general geometries will be:

$$N_s \approx \int ... \int_0^{\sigma+1} \sqrt{g} dx_1 ... dx_{n-1} dz \quad . \qquad \text{[S26]}$$

B. Specific cases of experimental interest.

B.1. 1-dimensional case. In this case, we recover the solution from (S11). According to the definition of the stem cell number given in equation (S26), leading to:

$$N_s^{1D} \approx 1 + \sqrt{\frac{k_r}{k_d}} \quad .$$

In two or more dimensions, the stem cell number does not come solely from the characteristic length set by fluctuations k_r/k_d along the direction of flow, but also from the geometry of the tissue.

B.2. Cylindric geometry with radius R. Direct application of equation (S26) on cylindric coordinates considering that the radius is R gives us:

$$N_s^{\text{cyl}} \approx 2\pi R \left(1 + \sqrt{\frac{k_r}{k_d}}\right) \quad .$$

We observe that this is equivalent to a 2-dimensional flat surface with $2\pi R$ cells at the bottom. Notice that this is true for the asymptotics, but not in the transient, since the neutral competition between cells at the same level will give rise to different speeds towards monoclonality depending on the radius R (5).

B.3. Two-dimensional hemispheric geometry with radius R. According to equation (S25), the stochastic fluctuations will project over the surface from the bottom pole to the cell located at:

$$z_s \approx 1 + \sqrt{\frac{\pi}{2}\frac{k_r}{k_d}} \quad .$$

The position of this cell will define a polar angle of:

$$\varphi_s \approx \frac{1}{R}\left(1 + \sqrt{\frac{\pi}{2}\frac{k_r}{k_d}}\right) \quad .$$

Therefore, any cell located below this angle from the center will be considered a stem cell, and the stem cell number will be given by equation (S26):

$$N_s^{2D} \approx 2\pi R^2 \left[1 - \cos\left\{\frac{1}{R}\left(1 + \sqrt{\frac{\pi}{2}\frac{k_r}{k_d}}\right)\right\}\right] \quad . \qquad \text{[S27]}$$

B.4. Spherical geometry. In this case, the probability of lineage survival in terms of distance from the center of the sphere will read:

$$p(c_n) \propto \exp\left\{-\frac{3k_d}{k_r}n^2\right\} \quad .$$

The variance is expected to be:

$$\sigma \sim \sqrt{\frac{k_r}{3k_d}} \quad .$$

In consequence, the effective stem cell number in a spherical system under the SCB dynamics taking place along the spherical coordinate can be approximated, according to equation (S26), to:

$$N_s^{\text{sphere}} \approx \frac{4}{3}\pi\left(1+\sqrt{\frac{k_r}{3k_d}}\right)^3 \quad .$$

C. A comment on tissue density, geometry and dynamical parameters. Two main assumptions underlie the previous computations. The first one is that the density of the tissue is considered constant, so that we can normalize un-ambiguously all lengths/surfaces/volumes by the characteristic cell size. If cell-cell dispersion occurs via cell-cell intercalations, then we find that density changes do not change the dynamics, as the dynamics is fully independent of cell size. However, if dispersal occurs as in kidney with extrusion and random cell motility, then cells randomly moving over the same distance would intercalate over "more" cells in a high density setting (introducing a length scale measured in absolute length units and not in units of cell size). Thus, density would increase stem cell number from a purely dynamical effect. More quantitatively, if k_r is given in terms of distance the cell moved in a time unit –instead of cell-cell intercalations–, then if the density f of the tissue is λf, with $\lambda > 0$, that means that there will be λ cells more for length/surface/volume unit. In that context, equation (S26) reads:

$$N_s \approx \int ... \int_0^{\lambda\sigma+1} \sqrt{g}dx_1...dx_{n-1}dz \quad ,$$

implying in the case e.g., of a hemispheric geometry, that:

$$N_s^{2D} \approx 2\pi R^2\left[1-\cos\left\{\frac{1}{R}\left(1+\sqrt{\lambda\frac{\pi}{2}\frac{k_r}{k_d}}\right)\right\}\right] \quad .$$

To have an idea of what does it mean quantitatively, if we have a radius of $R = 2$, as in the crypt, and $K_d = 1$, $k_r = 1$ in given distance units, $N_s^{2D} \approx 11$ cells. If the density doubles, i.e., $\lambda = 2$, then, according to the above equation $N_s^{2D} \approx 23$.

A second assumption is that we have considered the tissue geometry (for instance the length and width of mammary/kidney tips) to be constant. If geometry changes during the growth process, for example enlarging the radius of the bud, then the stem cell number would also change. The interplay between geometry and growing dynamics adds another layer of complexity to the SCB dynamics that goes beyond the scope of our current approach, and would be an interesting extension for the future.

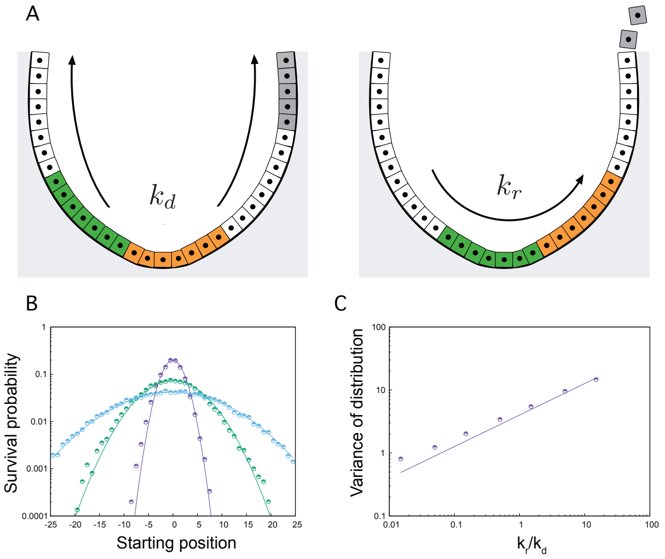

Fig. S5. A/ Schematic of the SCB dynamics where the source of stochasticity comes from "tectonic" movements: cell division can occur for every cell, which pushes all cells above, but repositioning relative to the bottom of the crypt/tip occur via global movements of the layer, at rate k_r (two clones shown competing before and after a movement). B/ Computational predictions of the 1-dimensional SCB dynamics in the presence of tectonic movements, with increasing rates k_r (purple to blue), in terms of survival probability as a function of starting position of the clone. All curves are very well fitted by normal distributions, as expected by our model. C/ Variance of survival probability as a function of starting position (i.e. functional stem cell number) as a function of the tectonic movements rates normalized by division rate k_r/k_d (dots), and theoretical prediction ($\sqrt{k_r/k_d}$, continuous line) from the SCB dynamics, showing that the overall behaviour of the system can be mapped to the one found with cell-cell random stochastic intercalations.

4. Noise in the stochastic conveyor-belt from "tectonic" epithelial movements

In this section, we explore an alternative source for noise in determining the number of functional stem cells, i.e. the possibility of global rearrangements of the epithelium relative to the optimal position (bottom of the crypt/edge of the tip –see Fig. (S5a) of this SI for a sketch. This is motivated by experiments in intestinal morphogenesis or mammary morphogenesis, where global three-dimensional bending of the epithelial modifies the location of the niche (see main text).

Importantly, performing full stochastic simulations of this process in one-dimensions revealed a strikingly similar paradigm compared to the version of the model introduced in the main text, with survival probabilities decaying as normal distributions away from the central, optimal position for survival – see Fig. (S3b) of this SI. Moreover, the variance of these probabilities, which define the number of functional stem cells, also scale as k_r/k_d as expected in the model from the main text –Fig. (S3c) of this SI.

This confirms that such tectonic movements can also be described in our coarse-grained model, simply renormalizing in long-term dynamics the intensity of the noise term k_r in the system (although one would expect tectonic movements to significantly change the short-term dynamics). Interestingly, this allows for the system to be "noisy", i.e. many functional stem cells to contribute to the long-term dynamics, without any clonal dispersion, showing that one must be careful in equating the two directly. This would in particular be relevant for the dynamics of intestinal crypts, where cells away from starting position 0 have been shown experimentally to still contribute long-term (see Fig. 2 of the main text), but where little clonal fragmentation was observed (raising the possibility that such tectonic collective movements could occur to reposition cells towards/away from the best location).

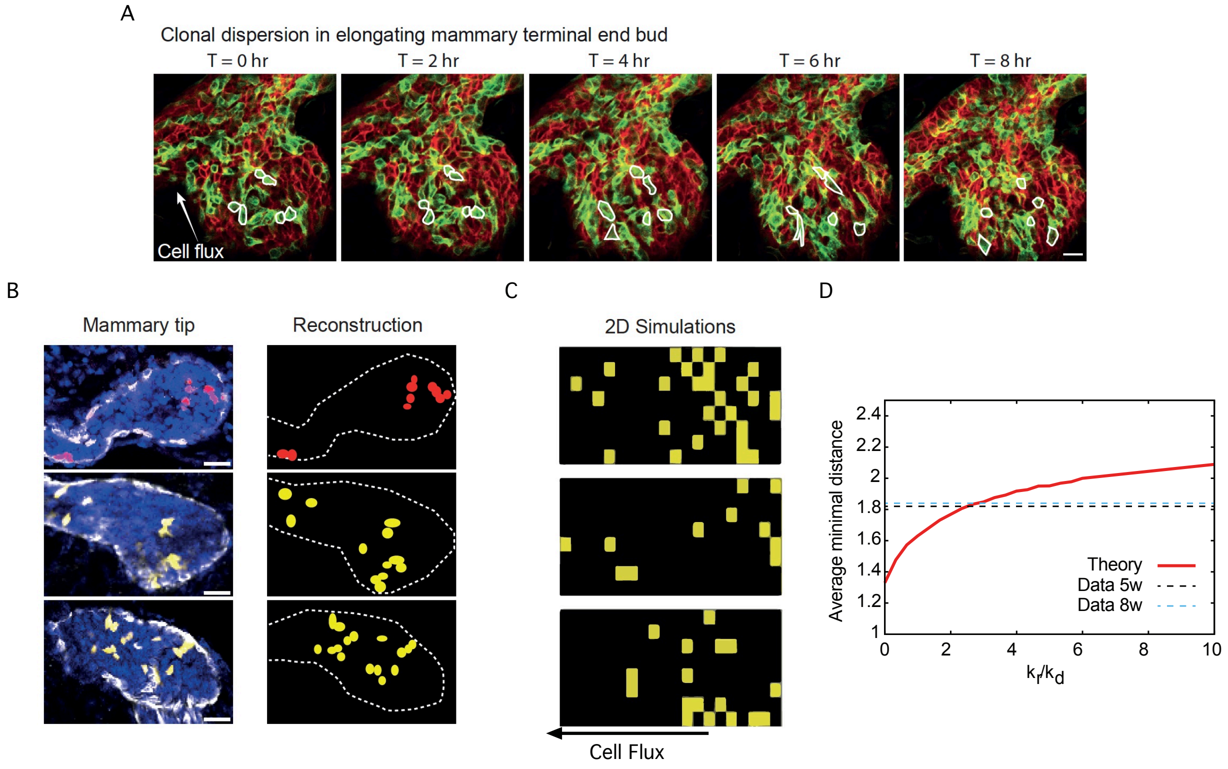

Fig. S6. Inferring k_r from clone dispersion in mammary gland development A/ Intravital microscopy images of a developing terminal end bud followed over multiple hours showing extensive cell rearrangements leading to clonal dispersion. Some cells are highlighted with white lines to illustrate the random cell movements within the terminal end bud. Scale bar represents 10 μm. B/ Confocal images of terminal end buds stained with keratin 14 (white) to label the basal cells, and DAP (blue) to label the nuclei. Cells from the same lineage are marked in red or yellow. The reconstruction of the mammary tips is shown in the right panel, from which the minimal distance between cells from the same lineage was inferred. Scale bar represents 25 μm. C-D/ Numerical simulations of clonal dispersion (three different outputs all with $k_r/k_d = 3$, panel C). The theoretical average minimal distance between cells in a clone increase in k_r/k_d (red line). The value where the expected nearest neighbour distance cross the real data (5w and 8w, dotted lines) is the estimated $k_r/k_d \approx 3$.

5. Numerical simulations and parameter estimation

A. 1-dimensional simulation. A one-dimensional array of 20 cells was initialised, where every cell was given an index $x = \{0, 1, ..., 19\}$ to identify their starting position in the crypt, corresponding to their lineage. 0 is the most advantageous position at the bottom of the crypt, and 19 is at the top where it will be removed from the crypt by any single division event below. The simulation parameters were k_d, the probability that a cell divides, and k_r, the probability that a cell switches positions with its neighbour in either direction. During the simulation, cells were chosen at random with equal probability to decide whether to divide or change neighbors. The simulation was terminated when the array was colonized by a single cell lineage –we denote this as a "win" by that lineage.

The probability of survival of a given lineage, $p(c_n)$ was calculated as the number of wins divided by the number of simulation runs. The simulation was repeated 2000 times. For every $\frac{k_r}{k_d}$, $p(c_n)$ was plotted as a function of the starting position n, and the data was fitted with the following function:

$$p(c_n) = \frac{1}{\sigma\sqrt{2\pi}}\exp\left\{-\frac{n^2}{2\sigma^2}\right\} \quad . \qquad \text{[S28]}$$

Consistently with the theoretical predictions given in equation (S11), we find that:

$$\sigma \sim \sqrt{\frac{k_r}{k_d}} \quad .$$

B. 2-dimensional simulation. A two-dimensional array of size 8x20 cells was initialised, where every cell was given an index $x = \{0, 1, ..., 19\}$ to identify their starting row in the crypt, corresponding to their lineage. 0 is the most advantageous row and 20 is the upper boundary after which cells are removed. Note that this boundary condition is largely irrelevant for the results because cells at these rows have vanishing chance to contribute to a winning lineage. Periodic boundary conditions were applied to every row (cylindrical geometry). This simulation uses the same parameters k_d and k_r, but now with a maximum of 4 possible neighbours for intercalation. The simulation was repeated 2000 times, and plotting the probability of survival of a given lineage shows that it fits to:

$$p_{2D}(c_n) = \frac{1}{\sigma\sqrt{2\pi}}\exp\left\{-\frac{n^2}{2\sigma^2}\right\} \quad . \qquad \text{[S29]}$$

In agreement with the theoretical predictions given in equation (S11), we find that:

$$\sigma \sim \sqrt{\frac{2k_r}{k_d}} \quad .$$

Both fits for intestinal homeostatic crypts and developing kidney shown in the main text were performed using this 2-dimensional simulation. In both cases, in order to build confidence intervals, in a non-parametric way, for our predictions of clonal survival as a function of position, we simulated 2000 times the number N of labelling events as in the data ($N = 45$ for intestine, $N = 24$ for kidney), and calculated the mean survival probability, as well as the 68% confidence interval around this prediction (i.e. 1 standard deviation around the mean). In both cases, we found that all of the experimental datapoint was contained within this interval. Finally, although on the long-term, survival probabilities converge towards a steady-state universal Gaussian distribution, the live-imaging datasets were experimentally acquired on finite timescales, requiring simulations to examine the dynamics of clonal conversion. We thus state below for each organ the duration of the simulations T (rescaled by the cell division rate k_d).

For the intestinal crypts, clones were initialized only at positions 0,1,2,3 and 4, to match with the experimental set-up where clones were only traced from Lgr5+ stem cells. Moreover, we defined a clone as "lost" in the system as soon as it didn't have any cells in this compartment (positions 0-4), again to match the way that the experimental data was recorded in Ref. (8). One should note that this assumption is expected to be largely irrelevant for our findings, given the results of Section 5: k_r/k_d in this system is small enough that $N_s < 5$, so that the probability for clones to come back in the Lgr5+ region after having left it during the early phase of clonal competition is vanishingly small. Plots in the main figure for intestinal crypts used the following parameters: $k_r/k_d = 1$ and a runtime of $Tk_d = 0.5$ (we note that the latter is relatively small, corresponding experimentally to a bit less than one full cell division in 3 days in Ref. (8), which could be linked to the method of intravital imaging (typical timescales reported in intestine are 1-2 days).

For the kidney tips, clones were initialized evenly in positions 0-10, which was also the definition for the compartment of clonal survival. Plots in the main figure used the following parameters: $k_r/k_d = 16$ (see main text for details on this parameter estimation) and a runtime of $Tk_d = 2$ (which is the typical average number of divisions seen in the experimental dataset during the time course, see Ref. (9)). The experimental data of Ref. (9) assigns to cells a starting position on a 10x10 grid, and notes whether a clone still remains in the tip at the end of the observation period. As position $(10, 10)$ was the edge of the tip, we calculated the euclidian distance of all coordinates (i, j) from position $(10, 10)$, which is the starting distance reported in Fig. 3 of the main text. One should note that because of the longer runtime of experiments, predictions in kidney are much-closer to their steady-state universal form.

C. 2-dimensional simulation and parameter estimation for fixed mammary gland samples. Finally, we also used these 2-dimensional simulations to fit the clonal fragmentation seen in mammary gland (where no long-term live-imaging was possible to follow clonal survival). To match experiments where cells were labelled in mouse of 3 weeks and collected at 5 weeks, and where the typical cell division rate is 16 hours, we used $Tk_d = 20$, although we found that the predictions were largely insensitive to this timing.

Given the low dose of clonal induction, all labelled cells in a tips of the same lineage (basal or luminal) can be considered to belong to a single clone. We thus experimentally measured for each labelled cell the distance to the closest labelled cell in a given tip, and built probability distributions for these nearest cell-cell distance across tips and mice (see details in section S6C) . Note that this was done in 2D projections, so that this experimental distance is a 2D approximation of the real 3D dispersion. We then performed the same computation for numerical simulations, for different values of k_r/k_d. As expected, for low values of k_r/k_d, cells are always close neighbours, so that the average cell-cell distance increased monotonously with k_r/k_d (plotted in Fig. S6D). Comparing this theoretical prediction to the experimentally measured average minimal distance then allowed us to estimate $k_r/k_d \approx 3$, although this remains a rough estimate, given the uncertainty in the measurements and the approximations made in estimating 2D distances. However, as shown in Fig. 4 of the main text, the full numerical probability distributions for the nearest cell-cell distance with $k_r/k_d = 3$ provided a satisfactory fit for the experimental data (both 5w and 8w), strengthening the approach. We also show in Fig. S6C different examples of 2D stochastic simulations for the output of the model with $k_r/k_d = 3$ (same parameter as in Fig. 4 of the main text) to give a better intuition of the variability of the clonal dispersion process.

6. Experimental procedures

A. Mice. All mice were females from a mixed background, housed under standard laboratory conditions, and received food and water ad libitum. All experiments were performed in accordance with the Animal Welfare Committee of the Royal Netherlands Academy of Arts and Sciences, The Netherlands.

B. Intravital microscopy. R26-CreERT2;R26-mTmG female mice were IP injected with 0.2mg/25gTamoxifen diluted in sunflower oil (Sigma) at 3 weeks of age to induce sporadic recombination in the developing mammary gland. At 5 weeks of age, a mammary imaging window was implanted near the 4th and 5th mammary gland –for details, see (10). Mice were anesthetized using isoflurane (1.5% isoflurane/medical air mixture) and placed in a facemask with a custom designed imaging box. Imaging was performed on an inverted Leica SP8 multiphoton microscope with a chameleon Vision-S (Coherent Inc., Santa Clare, CA, www.coherent.com), equipped with four HyD detectors: HyD1 (<455nm), HyD2 (455–490nm), HyD3 (500–550nm) and HyD4 (560–650nm). Collagen I (second harmonic generation) was excited with a wavelength of 860nm and detected with HyD1, GFP and Tomato were excited with a wavelength of 960nm and detected with HyD3 and HyD4. Mammary gland tips were imaged at an interval of 20-30 minutes using a Z-step size of 3μm over a minimum period of 8 hours. All images were acquired with a 25× (HCX IRAPO N.A. 0.95 WD 2.5mm) water objective.

C. Quantitative image analysis. Clonal dispersion in the developing mammary tips was measured in lineage traced whole mount glands from R26-CreERt2;R26-Confetti mice as previously described (11) (data contained both re-analysis of glands from this paper, as well as ones from the same experiments which had not been analyzed before). In brief, R26-CreERt2;R26-Confetti female mice were injected at 3 weeks of age with 0.2mg/25g Tamoxifen to achieve clonal density labelling (<1 cell per tip). Lineage traced mice were sacrificed at mid-puberty (5 weeks of age) or at the end of puberty (8 weeks). Mammary glands were dissected, fixed in periodate-lysing-paraformaldehyde (PLP) buffer (1% paraformaldehyde (PFA, Electron Microscopy Science), 0.01M sodium periodate, 0.075M L-lysine and 0.0375M P-buffer (0.081M Na2HPO4 and 0.019M NaH2PO4) (pH 7.4)) for 2 hours at room temperature (RT), and incubated for 2 hours in blocking buffer containing 1% bovine serum albumin (Roche Diagnostics), 5% normal goat serum (Monosan) and 0.8% Triton X-100 (Sigma-Aldrich) in PBS. Subsequently, glands were incubated with primary antibodies anti-K14 (rabbit, Covance, PRB155P, 1:700) or anti-E-cadherin (rat, eBioscience, 14-3249-82, 1:700), and secondary antibodies goat anti-rabbit or goat anti-rat, both conjugated to Alexa-647 (Life Technologies, A21245 and A21247 respectively, 1:400). Mammary glands were mounted on a microscopy slide with Vectashield hard set (H-1400, Vector Laboratories), and imaging was performed using a Leica TCS SP5 confocal microscope, equipped with a 405nm laser, an argon laser, a DPSS 561nm laser and a HeNe 633nm laser. All images were acquired with a 20x (HCX IRAPO N.A. 0.70 WD 0.5mm) dry objective using a Z-step size of 5μm (total Z-stack around 200μm). 3-dimensional tile scan images of whole-mount mammary glands were used to manually reconstruct the tips.

For n=106 tips in N=12 mammary glands derived from 6 mice (2 glands per mouse), labelled confetti cells were annotated in the schematic outline of the tips including information on the confetti colour for the mammary glands (GFP=green, YFP=yellow, RPF=red and CFP=cyan, see Fig. 4 of the main text and Fig. S6 for examples). The length and the width of the tips were measured, and the coordinates of each labelled confetti cell in the tip were determined. The coordinates were used to calculate the position of each labelled cell within the ductal tip, as well as the minimal distance to the nearest neighboring cells with the same confetti color (as a measure of the clonal dispersion within the stem cell zone).

References

1. C. W. Gardiner: (1983) *Handbook of Stochastic Methods for Physics, Chemistry and the Natural Sciences* Springer-Verlag:Berlin.

2. N. G. Van Kampen (2007) *Stochastic Processes in Physics and Chemistry* third edition, North Holland.
3. I. Karatzas, and S. E. Shreve (1988) *Brownian Motion and Stochastic Calculus* Springer-Verlag:Berlin.
4. C. Guillot,and T. Lecuit (2913) Mechanics of epithelial tissue homeostasis and morphogenesis *Science* 340, 1185–1189
5. C. López-García et al. (2010) Intestinal stem cell replacement follows a pattern of neutral drift. *Science* 330:822–825
6. W. Klingenberg (1978) *A course on differential geometry* Translated by D. Hoffman. Springer-Verlag:Berlin.
7. W. Kühnel (2006) *Differential Geometry: Curves–Surfaces–Manifolds* 2nd Edition. (Student Mathematical Library series, 77) American Mathematical Society:New york.
8. L. Ritsma et al. (2014) Intestinal crypt homeostasis revealed at single-stem-cell level by in vivo live imaging. *Nature*, 507:362–365
9. P. Riccio et al. (2016) Ret and Etv4 promote directed movements of progenitor cells during renal branching morphogenesis. *PLoS biology* 14.2: e1002382
10. M. Alieva et al. (2014) Imaging windows for long-term intravital imaging. IntraVital: 3, 2
11. Scheele C, et al. (2017) Identity and dynamics of mammary stem cells during branching morphogenesis. *Nature* 542(7641):313–317.

Supplementary Movie Legends

Movie S1. Numerical simulation of the stochastic conveyor belt model for $k_r/k_d = 0.5$ (initial position $n = 1$ wins).

Movie S2. Numerical simulation of the stochastic conveyor belt model for $k_r/k_d = 10$ (stills shown in Fig. 1D, initial position $n = 8$ wins)

Movie S3. Numerical simulation of the stochastic conveyor belt model for $k_r/k_d = 10$ (same parameters as Movie S2, although initial position $n = 2$ wins here)
