# Supplementary figures and images for: Stem cell lineage survival as a noisy competition for niche access
Source: Proc Natl Acad Sci U S A. 2020 Jul 1;117(29):16969–75. doi: 10.1073/pnas.1921205117 (PMC7382312; doi:10.1073/pnas.1921205117)

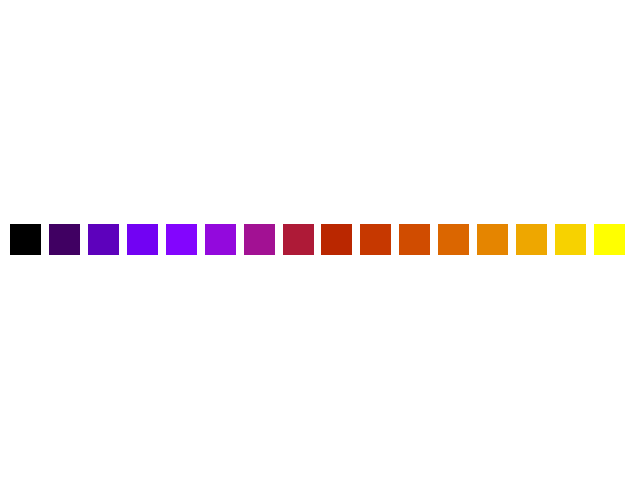

Supplement: Supplementary File [file pnas.1921205117.sm01.gif]

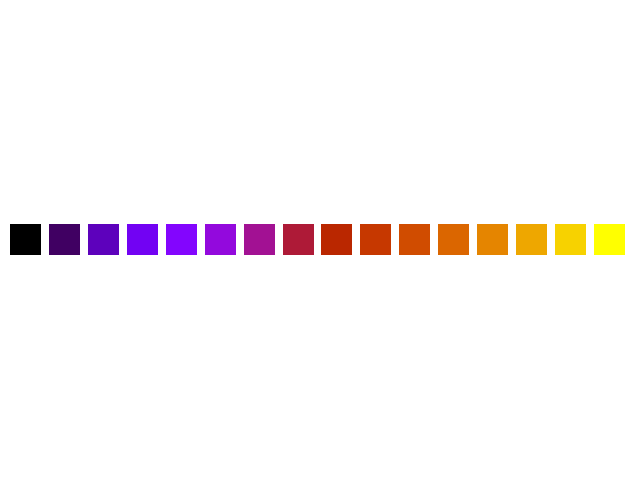

Supplement: Supplementary File [file pnas.1921205117.sm02.gif]

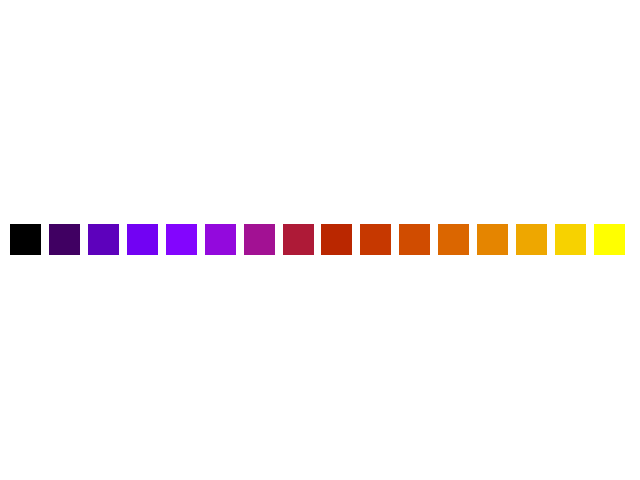

Supplement: Supplementary File [file pnas.1921205117.sm03.gif]
